# CXCR4 or CXCR7 antagonists treat endometriosis by reducing bone marrow cell trafficking

**DOI:** 10.1111/jcmm.14933

**Published:** 2020-01-06

**Authors:** Nicola Pluchino, Ramanaiah Mamillapalli, Shafiq Shaikh, Shutaro Habata, Aya Tal, Marie Gaye, Hugh S. Taylor

**Affiliations:** ^1^ Department of Obstetrics, Gynecology and Reproductive Sciences Yale School of Medicine New Haven CT USA

**Keywords:** AMD3100, BMDSC, bone marrow‐derived stem cells, CCX771, CXCR4, CXCR7, endometriosis

## Abstract

Adult stem cells have a major role in endometrial physiology, including remodelling and repair. However, they also have a critical role in the development and progression of endometriosis. Bone marrow‐derived stem cells engraft eutopic endometrium and endometriotic lesions, differentiating to both stromal and epithelial cell fates. Using a mouse bone marrow transplantation model, we show that bone marrow‐derived cells engrafting endometriosis express CXCR4 and CXCR7. Targeting either receptor by the administration of small molecule receptor antagonists AMD3100 or CCX771, respectively, reduced BM‐derived stem cell recruitment into endometriosis implants. Endometriosis lesion size was decreased compared to vehicle controls after treatment with each antagonist in both an early growth and established lesion treatment model. Endometriosis lesion size was not effected when the local effects of CXCL12 were abrogated using uterine‐specific CXCL12 null mice, suggesting an effect primarily on bone marrow cell migration rather than a direct endometrial effect. Antagonist treatment also decreased hallmarks of endometriosis physiopathology such as pro‐inflammatory cytokine production and vascularization. CXCR4 and CXCR7 antagonists are potential novel, non‐hormonal therapies for endometriosis.

## 1 INTRODUCTION

Endometriosis is a common, chronic inflammatory disorder affecting 5%‐15% of women in the reproductive age range.^1^, ^2^ It is characterized by the growth of endometrial tissue in ectopic locations, causing pain and infertility. In addition, endometriosis has a significant social and psychological impact on the lives of women, 63% of whom report serious negative impact on their quality of life.^3^, ^4^ The yearly costs of endometriosis treatment exceed $22 billion in the United States alone.^5^ There is currently no endometriosis specific treatment available. While surgery and medications for symptomatic pain relief are commonly used treatments, all current disease‐modifying medical therapies function by altering sex steroid hormones.^6^

Bone marrow‐derived stem cells (BMDSCs) are involved in the pathogenesis of the disease and contribute to the development of endometriotic lesions.^7^ BM‐derived stem cells participate in epithelial and stromal regeneration in endometrial tissue^8^ and endometriotic lesions,^9^, ^10^ and they are also likely the principal source of extra‐pelvic endometriosis.^7^, ^10^ Further, BM‐derived endothelial progenitor cells (EPCs) also contribute to endometriosis vascularization.^11^, ^12^ We have recently demonstrated a functional role of bone marrow‐derived stem cells in pregnancy.^13^

In cancer models, signals from the tumour microenvironment systemically stimulate quiescent bone marrow compartments, resulting in the expansion, mobilization and recruitment of BM‐derived cells^14^, ^15^ with a clear role in tumour development.^14^, ^16^ C‐X‐C motif chemokine ligand 12 (CXCL12) is one of the best‐characterized chemokines active in mobilization of BM‐derived stem cells in cancer and inflammation.^15^ In the endometriosis niche, CXCL12 participates in epithelial/glandular cell proliferation,^17^, ^18^ vasculogenesis and angiogenesis.^19^ CXCR4, a G protein‐coupled receptor (GPCR), is a receptor for CXCL12 expressed by bone marrow‐derived mesenchymal stem cells. CXCL12‐CXCR4 signalling is increased in women with endometriosis^20^, ^21^ and has been established as a crucial signal for BMDCs migration to endometriosis.^22^

CXCR7, also a GPCR, was recently identified as another receptor for CXCL12.^23^, ^24^ CXCR7 is phylogenetically closely related to other chemokine receptors and binds CXCL12 with a higher affinity than CXCR4, but it fails to couple to G proteins to induce typical chemokine receptor‐mediated cellular responses.^25^ CXCR7 has been demonstrated to regulate cell migration and survival through several pathways that include ligand scavenging, direct signal transduction and direct interaction with CXCR45.^26^, ^27^, ^28^ CXCR4 activation promotes tumour growth and metastasis in cancer,^29^, ^30^ and recent preclinical studies have shown similar effects for CXCR7.^28^, ^31^, ^32^ Agents blocking chemokine signalling by binding to these receptors are currently being developed for cancer therapy^27^, ^33^ (eg ClinicalTrials.gov identifier NCT02179970, NCT02737072), supporting an evaluation of their efficacy in endometriosis. Given the central role of the CXCL12/CXCR4/CXCR7 axis on BMDCs trafficking and in the pathogenesis of endometriosis, we hypothesized that blocking CXCR4 or CXCR7 in endometriosis using a reproductive and immune‐competent murine model would inhibit the growth of endometriosis. Both CXCR4 and CXCR7 may represent new targets for regulating BMDCs engraftment into endometriosis with potential therapeutic applications.

## 2 MATERIALS AND METHODS

### 2.1 Animals

C57BL/6J wild‐type and ubiquitin‐GFP mice were purchased from Charles River Laboratories and The Jackson Laboratory, respectively. Mice were housed and maintained (four to five per cage) in a room (21 ± 1C) with a 12‐hour light/dark cycle (7:00 am to 7:00 pm) with ad libitum access to food (Purina Chow; Purina Mills) and water, in the Yale Animal Resources Center (YARC) at Yale School of Medicine. Oestrous cyclicity was evaluated by vaginal cytology before endometriosis induction in order to transplant all tissue in dioestrus phase. In addition, oestrous cyclicity was evaluated during injection of vehicle or treatment to investigate any potential effect on the oestrous cycle. All animal experiments were conducted in accordance with approved protocols from Institutional Animal Care and Use Committee (IACUC) of Yale University. IACUC guidelines were clearly followed for animal care, 5FU treatments, bone marrow cell injections, blood collection and anaesthesia.

### 2.2 Conditional knockout of CXCL12 uterus of mice

Mice expressing Cre recombinase under control of the progesterone receptor (PGR) promoter were crossed to CXCL12*^fl/fl^* homozygotes (Jackson Laboratories stock numbers 017915 and 021773, respectively). Mice were genotyped to confirm targeted deletion of CXCL12 in PGR‐expressing tissues using PGR‐Cre specific primers (5′‐agttattgctgcccagttgc‐3′, 5′‐cccttctca tggagatctgtc‐3′, 5′‐gcgctaaggatgactctggtc‐3′) and CXCL12*^fl/fl^*‐specific primers (5′‐ctgcaccaggca gataatga‐3′, 5′‐tttggacaccagaaccttga‐3′). Uteri from PGR‐Cre+/CXCL12*^−^* ^/^ *^−^* and CXCL12*^fl/fl^* controls to be used for endometriosis induction (EI) were analysed for expression of total *CXCL12* transcript levels using the primer set 5′‐tgcccttcagattgttgcacg‐3′ and 5′‐ggctgttgtgcttacttgtttaaagc‐3′, with GAPDH primers 5′‐gcctgcttcaccaccttctt‐3′ and 5′‐atggccttccgtgttcctac‐3′. Uteri from CXCL12*^fl/fl^* or PGR‐Cre+/CXCL12*^−^* ^/^ *^−^* mice were sutured onto cycling wild‐type females (n = 4 and n = 10 hosts, respectively). Four weeks after EI, lesions were extracted, and total lesion area was measured using ImageJ software after subtracting cyst area. Mean ± standard error of the mean (SEM) was calculated for the various experiments using GraphPad Prism 6 (GraphPad Software). An unpaired *t* test was used to compare lesion size in the two groups.

### 2.3 BM conditioning and transplantation

Six‐week‐old female C57BL/6J wild‐type mice received 125 mg/kg of 5‐FU by i.p injections 6 days and 1 day before bone marrow transplantation (BMT). In addition, stem cell factor (SCF, 50 mg/kg) was injected i.p twice before BMT, as we have previously described.^34^ Transplantation of fresh BM cells was performed as described previously.^9^ Briefly, bone marrow cells were obtained from 6‐ to 10‐week‐old C57BL/6J ubiquitin‐GFP male donor mice by flushing the marrow from femurs and tibias into cold sterile PBS and filtered through 70‐μm cell strainer (BD Biosciences, San Jose, CA, USA). The yield and viability of BM cells were determined by trypan blue staining. Next, 20 × 10^6^ unfractionated BM cells were iv injected to recipients 6 days after the beginning of BM conditioning. Lesions were stained for Ki‐67 proliferation marker as described below.

### 2.4 Induction of endometriosis in mice

Endometriosis in mice was surgically induced under aseptic conditions and anaesthesia using a modified method previously described.^10^, ^35^ Surgery was performed 30 days following BMT. Uterine horns were removed from wild‐type female donor mice at dioestrus (low oestrogen stage), opened longitudinally, cut into fragments of 3‐mm and transplanted onto the peritoneal wall of recipient mice by suturing. Three uterus fragments from wild‐type mice as well as CXCL12^−/−^ were systematically transplanted into peritoneal wall of each mouse. After treatments, ectopic lesions were collected. Ectopic lesion volume was calculated as a half ellipsoid that approximated lesion shape on the peritoneum, using formula V = (1/2) (4/3)πr1^2^r2 (r1 and r2 are radii, r1 < r2).^36^ Sham mice were subjected to the surgery, but in place of suturing uterine tissue, peritoneal tissue from the ventral midline was used. Sham mice did not receive any treatment.

### 2.5 Endometrial cell culture

Human endometrial tissue was obtained from consenting subjects with a diagnosis of endometriosis under a protocol approved by the Yale Institutional Review Board (IRB). Primary cell cultures were prepared from the tissue collected from endometrium of endometriosis patients (n = 7). The finely minced tissue was incubated in Hanks balanced salt solution containing 4‐(2‐hydroxyethyl)‐1‐piperazineethanesulfonic acid (HEPES; 25 mm), 1% penicillin/streptomycin, collagenase (1 mg/mL, 15 U/mg) and DNase (0.1 mg/mL, 1500 U/mg) for 45 minutes at 37_C with agitation. During and at the end of the incubation, the tissue was pipetted gently to disperse the cells every 15 minutes. Endometrial cells were pelleted, washed and suspended in Ham's Dulbecco's modified Eagle medium: nutrient mixture F‐12 (DMEM/F12) (1:1) containing 10% foetal bovine serum (FBS), 1% penicillin/ streptomycin and 1% amphotericin B. A mixture of endometrial cells (epithelial and stromal) was passed through a 40‐mm sieve, which allowed stromal cells to pass through while epithelial cells are retained on the sieve (Millipore, Billerica, Massachusetts). The stromal cells were cultured and treated with AMD3100 (25 µg/mL) at 50% of cell confluence and cell proliferation determined by counting the number of cells on day 1 and day 6. All the experiments were carried out three times, each in duplicate. Untreated cell count on day 1 and day 6 taken 100%.

### 2.6 In vivo CCX771 and AMD3100 treatment

The ligand CCX771 (30 mg/kg body weight) kindly provided by ChemoCentryx (Mountain View, CA, USA) or vehicle (10% Captisol) was administered daily (s.c) in 100 μL vehicle throughout the treatment period. AMD3100 (5 mg/kg) from Sigma‐Aldrich was administered daily (s.c) in 100 μL vehicle.^37^

### 2.7 Flow cytometry analysis of murine endometriosis

Endometriosis lesions were removed, finely minced and subsequently digested with a solution of Hanks' balanced salt solution (Life Technologies Inc) containing HEPES (25 mmol/L), collagenase B (1 mg/mL; Roche Diagnostics) and deoxyribonuclease I (0.1 mg/mL; Sigma‐Aldrich) for 60‐90 minutes at 37°C. All samples were filtered through 70‐micron mesh and centrifuged at 300 *g* at 4°C for 5 minutes. Cell suspensions were stained with fluorescently conjugated antibodies to CD45 (catalog #103116; BioLegend). Flow cytometry was performed on a fluorescence‐activated cell sorting Beckman Coulter MoFlo machine (Beckman Coulter) using the corresponding excitation wavelength for GFP and CD45. Gates were applied to forward scatter/side scatter dot plots to exclude non‐viable cells and cell debris. Data were analysed using the software FlowJo V10 (FlowJo).

### 2.8 Immunohistochemistry and immunofluorescence

Tissue from endometriotic lesions was fixed in 4% paraformaldehyde and embedded in paraffin. Five‐micrometre tissue sections were mounted on slides followed by 10 minutes boiling in sodium citrate (pH 6) for antigen retrieval, and blocking using 10% serum (Vector Laboratories). Tissue sections on slides were incubated at 4°C overnight with anti‐CXCR7 primary antibody (catalog #MAB42273‐SP; Clone 11G8, 1:1000; R & D Systems) or anti‐CD31 (catalog #ab28364; 1:300) or anti‐Ki‐67 (catalog #ab15580; 1:1000; Abcam) followed by 30 minutes at room temperature with appropriate biotinylated secondary antibody (1:200; Vector Laboratories), and detection used ABC Vectastain Elite reagents with DAB plus H_2_O_2_ (catalog #SK‐4105; Vector Laboratories). Tissue sections were counterstained with haematoxylin (Sigma‐Aldrich). Images of stained sections were captured using Olympus BX‐51 microscope (Olympus). For colocalization of GFP‐positive BMDCs by immunofluorescence, sections were incubated with the following primary antibodies purchased from Abcam: polyclonal anti‐GFP antibody (catalog #ab6673; 1:1000), anti‐CD45 (catalog #ab25386; 1:300), anti‐Sca‐1 (catalog #701919, Invitrogen; 1:400), anti‐CD31 (1:200), anti‐CXCR4 (catalog #PA3‐305; 1:300), anti‐CXCR7 (1:500, # MAB42273, R&D Systems), anti‐CXCL12 (catalog #ab18919; 1:300) and anti‐F4/80 (catalog #ab111101; 1:160). Secondary antibodies used were Alexa Fluor 568‐conjugated donkey anti‐goat (catalog #A11057) and Alexa Fluor 488‐conjugated donkey anti‐rabbit (catalog #A21206) or Alexa Fluor 647‐conjugated donkey anti‐rat (catalog #ab150155) or Alexa Fluor 488‐conjugated donkey anti‐rat (catalog #A21208) or Alexa Fluor 488‐conjugated goat antimouse (catalog #A28175), all in 1:200 dilution (Life Technologies). Sections were mounted under coverslips using Vectashield fluorescent mounting media with 46‐diamidino‐2‐phenylindole (DAPI) (catalog #H‐1200; Vector Laboratories.) Isotype controls were used to confirm the specificity of CXCR4, CXCR7 and CXCL12 by immunofluorescence studies with rabbit IgG antibody (Abcam, #37415) for CXCR4 and CXCL12 or mouse IgG1 (# MAB002, R & D Systems) shown in Figure S1. Visualization of the slides was performed using a laser scanning confocal microscope (LSM 710; Zeiss) and the ZEN software (Carl Zeiss). Stained cells were quantified by monitoring the average numbers of positively stained cells relative to the total number of cells from six randomly chosen fields.

### 2.9 Apoptosis assays

The TUNEL assay was performed to assess the apoptosis activity in ectopic lesions sections with the In situ Apoptosis Detection Kit (Abcam, catalog #ab206386) according to the manufacturer's instructions. The TUNEL reaction preferentially labels DNA strand breaks generated during apoptosis and allows discrimination of apoptosis from necrosis. In this assay, terminal deoxynucleotidyl transferase (TdT) binds to exposed 3′‐OH ends of DNA and are detected using a anti‐digoxigenin‐conjugated HRP. Diaminobenzidine (DAB) reacts with the HRP‐labelled sample to generate an insoluble coloured (brown) substrate at the site of DNA fragmentation. Briefly, deparaffinized and rehydrated ectopic lesion tissue sections were permeabilized using protease K (20 μg/mL, Sigma) for 30 minutes at room temperature and endogenous peroxidases were inactivated with 3% H_2_O_2_ for 10 minutes at room temperature. After two washes with immunoassay buffer, sections were incubated with TdT enzyme for 2 hours and the reaction was stopped with blocking buffer for 10 minutes and then incubated with anti‐digoxigenin‐conjugated HRP and diaminobenzidine (DAB) solution for 30 and 20 minutes, respectively. DAB reacts with the HRP‐labelled sample to generate an insoluble coloured (brown) substrate at the site of DNA fragmentation. Sections were dehydrated and placed cover slips with fluorescent mounting medium. All washes between each step were performed with buffer. Slides were analysed with fluorescence microscopy. (Leica, DM 5000B; Leica CTR 5000; Germany).

### 2.10 Quantitative real‐time polymerase chain reaction (qRT‐PCR)

Total RNA was isolated from endometriotic lesions by TRIzol reagent (Life Technologies) followed by purification using Quiagen cleaning kit (Valencia, CA, USA) to prepare cDNA with 50 ng RNA in a 20 μL reaction mixture by iScript cDNA Synthesis Kit (Bio‐Rad Laboratories). Quantitative real‐time PCR was performed to quantify gene expression using specific primers and SYBR Green (Bio‐Rad) and optimized in the MyiQ Single Color Real‐Time PCR Detection System (Bio‐Rad). The specificity of the amplified transcript and absence of primer‐dimers was confirmed by a melting curve analysis. Gene expression was normalized to the expression of β‐actin for each sample. Relative mRNA expression for each gene was calculated using the comparative cycle threshold (Ct) method, also known as the 2^−ΔΔC(T)^ method.^38^ All experiments were carried out in triplicate and nuclease‐free water was used as a negative control replacing the cDNA template.

### 2.11 Statistical analysis

Data were analysed using GraphPad Prism 7.0 (GraphPad Software Inc). An unpaired Student's *t* test for percentage of labelled cells (PLC) or one‐way ANOVA for cell counts and RT‐PCR data was used to determine statistical significance. Data are expressed as means ± standard error (SE).

## 3 RESULTS

### 3.1 BMDSCs engrafting endometriosis express CXCR4, CXCR7 and stem cell markers

To characterize BMDSCs engrafting endometriosis in vivo, we used a 5‐FU‐based submyeloablation mouse bone marrow transplantation model according to our previously published protocol.^34^ This method results in efficient bone marrow donor chimerism (approximately 50% donor‐derived cells) while preserving ovarian function and fertility. Previous studies on BMDCs trafficking in endometriosis have relied on sub‐lethal irradiation as conditioning for BMT with resulting loss of ovarian function, oestrous cyclicity,^9^, ^11^, ^39^, ^40^ dysfunctional immune signalling^41^ and blood flow.^42^ Preserving gonadal activity is crucial because models of murine endometriosis using reproductive and immuno‐competent mice show the same endocrine features of human disease, namely aberrant oestrogen signalling and progesterone resistance.^43^, ^44^ Moreover, gonadal steroids regulate the CXCL12‐CXCR4 axis in endometrium and endometriosis.^45^, ^46^ Thus, BMT from GFP‐positive donors allowed the investigation of the contribution of BMDCs mobilization and engraftment in endometriosis. We have previously demonstrated that GFP+ BM cells contain an immune population derived from haematopoietic system cells that are CD45+. As haematopoietic cells, all immune cells originate from the bone marrow, and the extensive contribution of immune in endometriosis pathophysiology has been documented. Further, CD45‐ bone marrow‐derived cells in mice are a heterogenous population that includes BM stromal cells, endothelial progenitor cells and mesenchymal stem cells^47^ and are identified by their surface markers.^48^, ^49^ CD45‐ cells of BM origin contribute to endometriosis development. Here we observed the engraftment of bone marrow‐derived stem cells (BMDSCs), characterized as CD45‐/GFP+/Sca1+, to endometriotic lesions as shown in Figure S2. While characterized here by immune staining alone, we have previously shown that these engrafted cells can give rise to adipogenic, osteogenic and chondrogenic lineages.^47^

**Figure 1:**
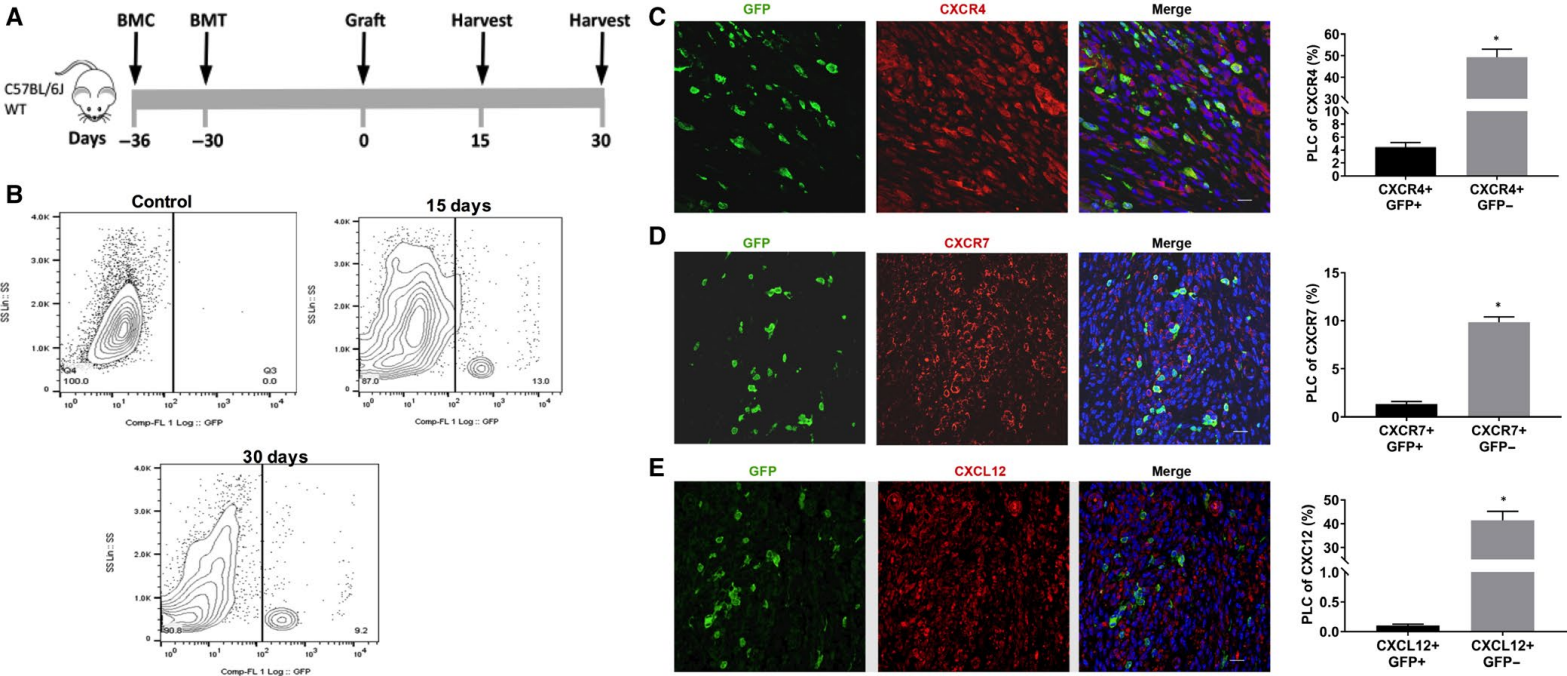
A model of 5‐FU submyeloablation to characterize the bone marrow cell trafficking into endometriotic lesions in mice. Schematic model: Treatment for bone marrow conditioning (BMC) started on day −36 before uterine fragment grafting and 6 days before bone marrow transplantation (BMT) (A). Ectopic lesions were harvested 15 and 30 days (each group n = 6) after grafting uterine fragments into the peritoneal. Representative images of flow cytometry analysis of ectopic lesion cells on day 15 and 30 after uterine fragment grafting demonstrating the percentage of GFP‐positive cells in endometriosis. B, Control group (n = 6 mice), represented by GFP‐negative endometriosis lesions, was used to set a gate. Fluorescence confocal microscopy analysis of mouse endometriosis sections (C, D and E). Representative images of IF and quantitative analyses of cells expressing CXCR4, CXCR7 or CXCL12 and GFP, as a per cent of total endometriosis cells. Murine lesions were stained with anti‐GFP antibody (green) and co‐stained with CXCR4 antibody (red) (C), with CXCR7 antibody (red) (D) and with CXCL12 antibody (red) (E). Nuclei were stained by DAPI and are shown in blue. BMC, Bone morrow conditioning; BMT, Bone morrow transplantation; PLC, percentage of labelled cells. Results shown as mean ± SE. *P < .05 vs vehicle. Scale bar: 20 μm

**Figure 2:**
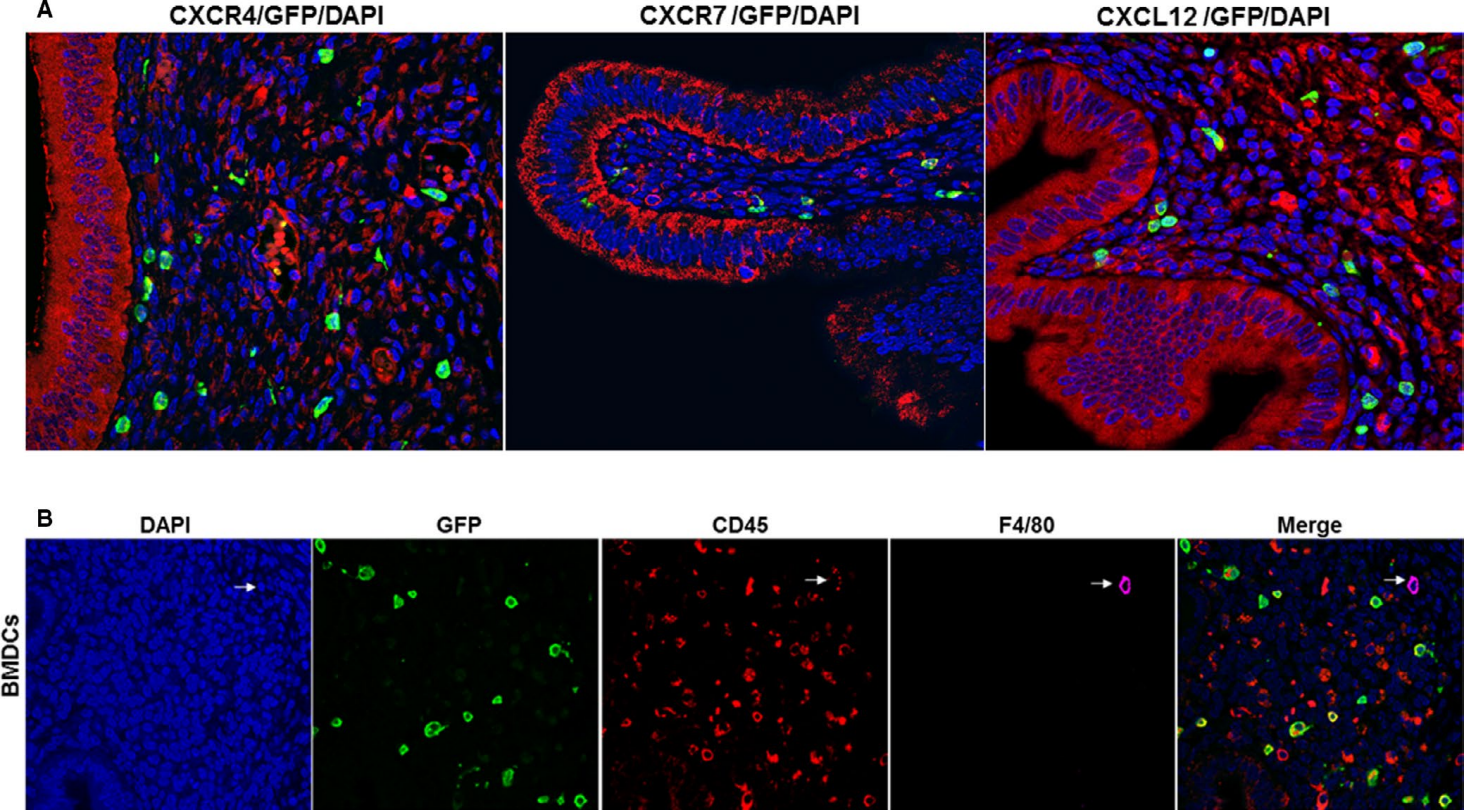
Immunostaining of CXCR4, CXCR7 and CXCL12 and BMDCs. A, Representative immunofluorescence images of CXCR4, CXCR7 and CXCL12 staining in the epithelial cells of murine endometriosis. In our model, no GFP (bone marrow‐derived cells) was identified in the epithelium. Scale bar: 10 μm. B, Engrafted BMDCs express CD45 and F4/80. BMDCs: Engrafted bone marrow‐derived immune cells (BMDCs) were stained with DAPI or immunofluorescence for CD45 and F4/80. Engrafted BMDCs shows the presence of non‐immune (CD45 negative) and immune cells (CD45 positive), some of which co‐express F4/80, a marker of macrophages. Included is a cell that is a bone marrow‐derived (GFP+), CD45+, F4/80+ macrophage (arrow) Scale bar: 10 μm

Treatment for bone marrow conditioning (BMC) started on day −36 before uterine fragment grafting and 6 days before bone marrow transplantation (BMT) as shown in (Figure 1A). Flow cytometry analysis of single cell suspensions from endometriotic lesions, 15 and 30 days after BMT, demonstrated no significant difference in the number of GFP + BMDCs between 15 days (11.5 ± 2.4%) and 30 days (10.3 ± 2.1, *P* < .05 vs day 15) (Figure 1B). Bone marrow (BM)‐derived cells are known to express chemokine receptors^50^ including CXCR4^16^ Using immunofluorescence, we revealed that in endometriosis CXCR4 labelled up to 50% of the total endometriosis cell population (Figure 1C). To identify bone marrow‐derived MSCs expressing CXCR4, we counted GFP/CXCR4 double‐positive cells; BM‐derived GFP+ cells that colocalized with CXCR4 represented 4.4 ± 0.7% of total cells (Figure 1C); however, a substantial number (approximately 15%) of BM‐derived cells did not express CXCR4 at the time of assay. In contrast, only 1.4 ± 0.7% of total cells showed colocalization of GFP with CXCR7, although up to 11% of total endometriosis stromal cells expressed CXCR7 (Figure 1D). Remarkably, a small percentage of BMDCs engrafting endometriotic lesions expressed CXCL12 (0.1 ± 0.02% of total cells) (Figure 1E). No GFP (bone marrow‐derived cells) was found in the epithelium (Figure 2A). Also, we noticed that some engrafted bone marrow‐derived cells were positive for CD45 and some of them were simultaneously positive for F4/80, a marker for macrophages (Figure 2B).

### 3.2 Pharmacological antagonism of CXCR4 or CXCR7 reduced the engrafting of BMDSCs into endometriosis

**Figure 3:**
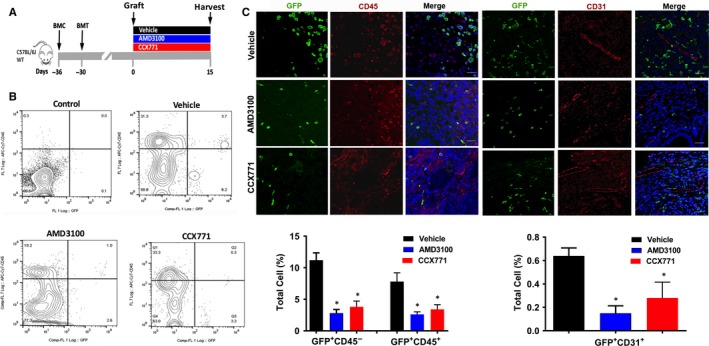
Targeting CXCR4 or CXCR7 reduced BMDCs engraftment of endometriosis. Schematic model: treatment for bone marrow conditioning (BMC) started on day −36 before grafting uterine fragments and 6 days before bone marrow transplantation (BMT) (A). Treatment with AMD3100, CCX771 or vehicle (three groups, each group n = 6 mice) started on day 1 after grafting uterine fragments into the peritoneal cavity (day 0). Ectopic lesions were harvested after 15 days of treatment. B, Representative images of flow cytometry analysis of ectopic lesion cells after treatment showing the percentage of GFP‐positive cells in endometriotic lesions. Control, represented by GFP‐negative endometriosis lesion stained with CD45 isotype, was used to set gates (B). Fluorescence confocal microscopy analysis of mouse endometriosis sections. Representative images of IF and quantitative analyses of cells expressing CD45 (C) or CD31 (D) and GFP, as a per cent of total cells, following treatment. Nuclei were stained by DAPI and are shown in blue. PLC, percentage of labelled cells. BMC, Bone morrow conditioning. BMT: Bone morrow transplantation. Results shown as mean ± SE. *P < .05 vs vehicle. Scale bar: 20 μm

The high expression of CXCL12 in endometriosis and expression of CXCR4 and CXCR7 in BMDCs suggested that CXCL12 might serve to regulate BMDCs trafficking towards endometriosis. To test this hypothesis in our murine model, we administered the small molecule CCX771, a CXCR7 antagonist,^51^ AMD3100 (Plerixafor), a CXCR4 antagonist, or vehicle control for 15 days (day 1‐15 after graft) (Figure 3A). We showed that of total endometriosis cells, the majority of BMDCs engrafting endometriosis were CD45^−^ compared to CD45+ cells (leucocytes) as shown in Figure 3B. AMD3100 significantly reduced the engraftment of GFP+ cells in endometriosis including GFP + CD45^+^ leucocytes and GFP + CD45^−^ BMDSCs, consistent with a reduction of recruitment of total BMDCs in the lesions. Similarly, CCX771 reduced total GFP^+^ cells significantly in endometriosis including GFP^+^CD45^+^ and GFP^+^CD45^−^ cells (Figure 3C). Taken together, these findings demonstrate that targeting CXCR4 or CXCR7 reduced BMDCs engraftment, with a marked predominance for BM cells not expressing the pan‐leucocyte protein CD45. Blocking CXCL12 activity on its receptor greatly reduced BMDCs engraftment in endometriosis.

To further characterize BMDCs affected by AMD3100 and CXC771 treatment, we investigated the endometriosis engraftment of BM‐derived endothelial cells expressing CD31. We confirmed the contribution of BM to endometriosis vascularization as previously reported.^52^ Specifically, although a small percentage of GFP^+^CD31^+^ cells engrafted endometriosis (0.6 ± 0.1%), the presence of these cells in murine endometriosis was significantly reduced after treatment with either AMD3100 (0.18 ± 0.04%) or CCX771 (0.3 ± 0.1%) (Figure 3D).

### 3.3 Pharmacological antagonism of CXCR4 or CXCR7 led to the regression of endometriosis

**Figure 4:**
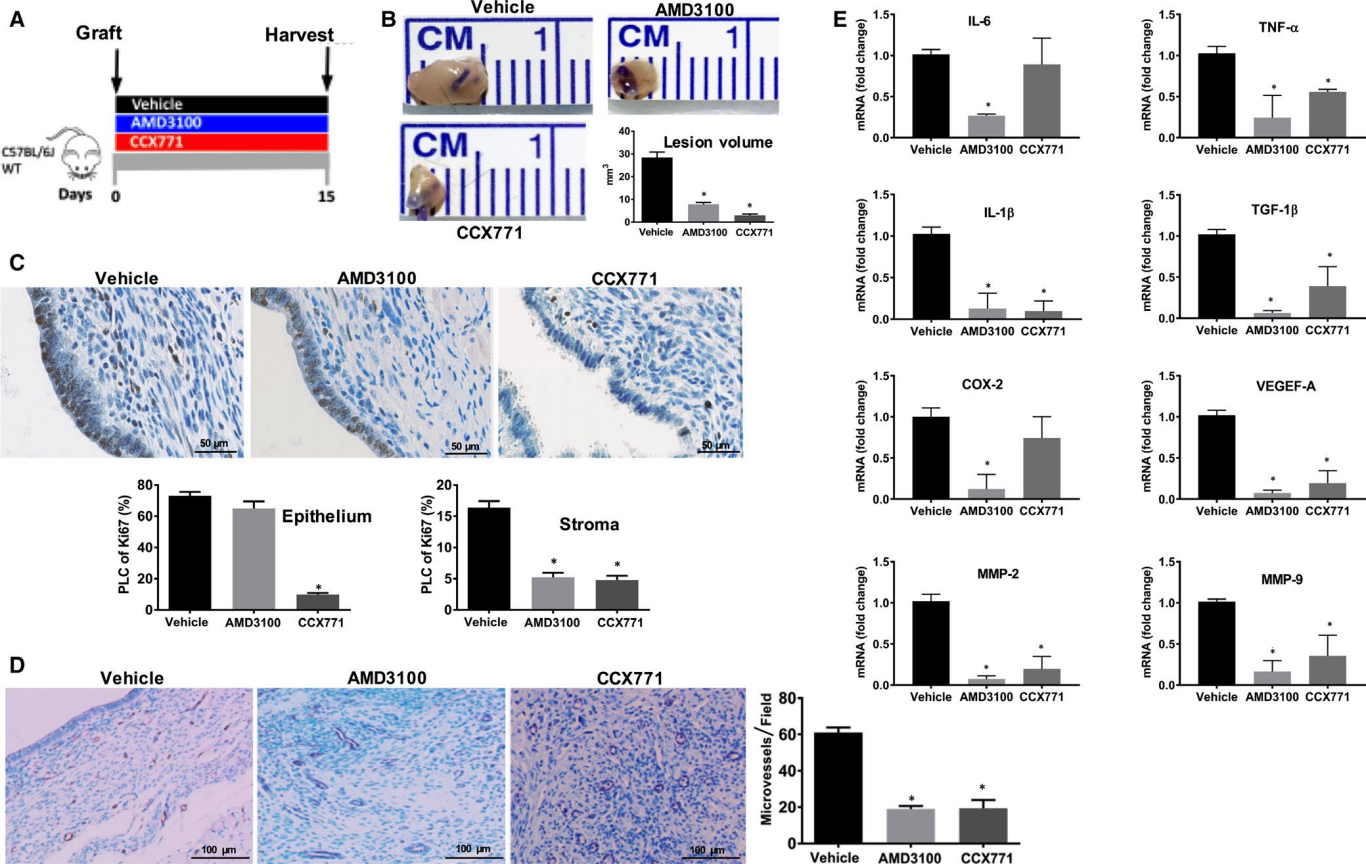
Targeting CXCR4 or CXCR7 reduced endometriosis development. Early growth model: AMD3100 or CCX771 or vehicle (three groups, each group n = 6 mice) injection started on day 1 after uterine fragments into the peritoneal cavity (day 0) of wild‐type animals (A). Ectopic lesions were harvested after 15 days of treatment. Representative pictures of ectopic lesions isolated from wild‐type mice with endometriosis subcutaneously treated with either vehicle or receptor antagonists (B). Lesion volume measurement after 15 days of treatment. IHC and quantitative analyses of the expression patterns of Ki‐67 in epithelial or stromal cells of ectopic lesions of wild‐type mice after treatment (C). Scale bar: 50 μm. IHC, and quantification of microvessel density of ectopic lesions of wild‐type mice treated with ligands or vehicle using CD31 staining (D). Scale bar: 100 μm. Expression of IL‐6, TNF‐α, IL‐1β, TGF‐β, COX‐2, VEGEF‐A and MMP2, MMP9 in ectopic lesions was analysed by quantitative reverse transcription PCR (qRT‐PCR) after 15 days of treatment. mRNA levels are expressed relative to transcript level in ectopic lesions of mice treated with vehicle (E), set at 1.0. Results showed as mean ± SE. *P < .05 vs vehicle

To investigate whether targeting CXCR4 or CXCR7 could have clinical application, we examined volumetric and molecular changes of endometriotic lesions following treatment. We first used an early growth model (treatment over days 1‐15 after graft placement) to examine the effectiveness of AMD3100 and CCX771 in preventing ectopic lesion establishment. (Figure 4A). Either AMD3100 or CCX771 treatment prevented the development of endometriosis following the engraftment of donor uteri fragments in normal cycling mice. Treatment with AMD3100 or CCX771 resulted in more than a 50% reduction of the lesion volume after 15 days comparing either agent to vehicle treated controls. There were no significant differences between the two active treatments (Figure 4B). IHC labelling confirmed reduction in Ki‐67 expression in the stroma following both treatments whereas only CCX771 reduced epithelial Ki‐67 expression (Figure 4C). Both treatments reduced the vessel density by 40% in endometriosis determined by IHC staining (Figure 4D). Treatment was also associated with a decline in mRNA expression of several pro‐inflammatory cytokines including IL‐6, TNF‐α, IL‐1β and TGF‐β, known to be highly expressed in endometriosis. Both treatments reduced the mRNA expression of vascular endothelial growth factor A (VEGF‐A) and matrix metalloproteinase MMP2 and MMP9. CCX771 had no effect on IL‐6 or COX‐2 expression in this model (Figure 4E).

**Figure 5:**
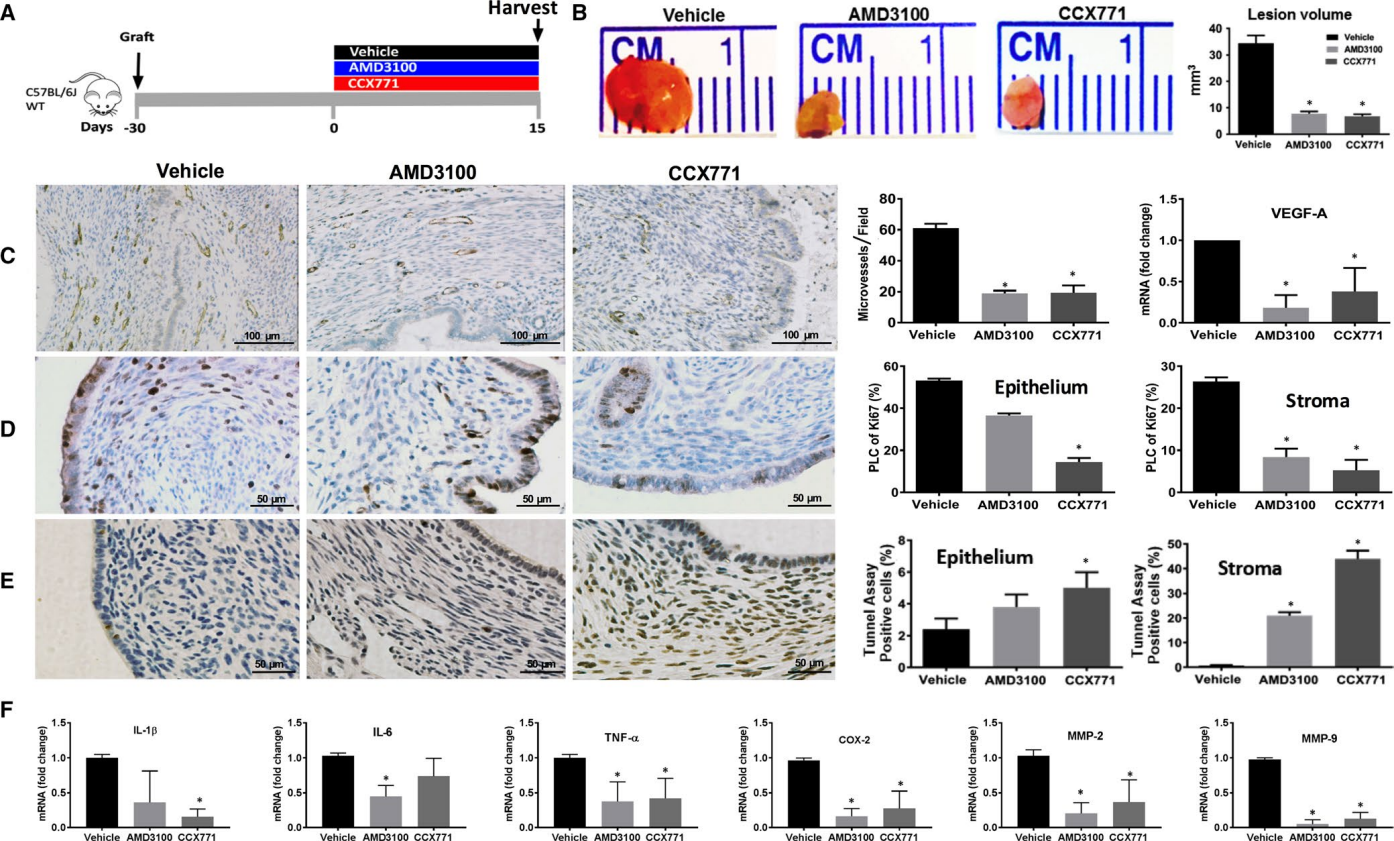
Targeting CXCR4 or CXCR7 induced the regression of endometriosis. Treatment model: AMD3100 or CCX771 or vehicle was subcutaneously injected into three groups of mice separately (each group n = 6 mice) of mice starting on day 15 after grafting uterine fragment into the peritoneal cavity (day 0) of wild‐type animals (A). Ectopic lesions were harvested after 15 days of treatment (day 30). Representative pictures of ectopic lesions isolated from wild‐type mice with endometriosis subcutaneously treated with either vehicle or receptor antagonists (B). Lesion volume measurement after 15 days of treatment. IHC, and quantification of microvessel density of ectopic lesions of wild‐type mice treated with ligands or vehicle using CD31 staining (C). Scale bar: 100 μm. IHC and quantitative analyses of the expression patterns of Ki‐67 in epithelial or stromal cells of ectopic lesions of wild‐type mice after treatment (D). Scale bar: 50 μm. Representative pictures and quantification of deoxynucleotidyl transferase‐mediated deoxyuridine triphosphate nick end labelling (TUNEL) assays in epithelial and stroma cells (E) (the number of TUNEL‐positive stained cells are shown in the bar graphs as percentage to the total cells). Scale bar: 50 μm. Expression of IL‐1β, IL‐6, TNF‐α, COX‐2 and MMP‐2 and MMP‐9 in ectopic lesions was analysed by quantitative reverse transcription PCR (RT‐PCR) after 15 days of treatment (F). mRNA levels are expressed relative to transcript level in ectopic lesions of mice treated with vehicle, set at 1.0. Results show mean ± SE. *P < .05 vs vehicle

In order to further test our hypothesis, treatment with AMD3100 and CCX771 was started after ectopic lesions were already established in recipient mice (treatment over days 15‐30 after graft placement) (Figure 5A). This more closely models the pre‐existing lesions found in humans at the time of endometriosis diagnosis. Both treatments reduced the vessel density in endometriosis determined by IHC staining and VEGF‐A mRNA expression (Figure 5C). Consistent with the results presented above, both AMD3100 and CCX771 reduced endometriosis lesion volume by 60% (Figure 5B) and cell proliferation by 70% in the stromal compartment as determined by ki‐67 staining (Figure 5D). Both treatments also increased apoptosis as determined by TUNEL assay (Figure 5E). CCX771 treatment increased the apoptosis significantly both in stroma and epithelium while AMD3100 reduced only stromal apoptosis. Moreover, the administration of either drug (d15‐d30 after graft) reduced the expression of pro‐inflammatory cytokines such as IL‐1β, IL‐6 and TNF‐α, COX‐2 and invasion pathway proteins MMP2 and MMP9 as shown in Figure 5F, confirming our hypothesis that CXCR4‐CXCR7 may treat endometriosis and endometriosis‐associated inflammation.

In addition, the oestrous cycle was evaluated during injection of vehicle, AMD3100 and CCX771, to investigate any potential effects on mice oestrous. Phase identification was performed by vaginal cytology. No significant changes in the length of each oestrous cycle stage were recorded in animals receiving either drug, supporting the absence of systemic endocrine effects and demonstrating that the effects of these agents worked thorough a hormone independent pathway targeting stem cell recruitment.

### 3.4 No significant change in lesion size in endometriosis from uterine‐specific CXCL12 null mice

To assure that the effects of treatment was due to blockade of BMDC recruitment and not local effects of endometrial cell‐derived CXCL12, we repeated these experiments creating endometriosis derived from uterine‐specific CXCL12 null mice. Uteri of PGR‐Cre+/CXCL12*^−^* ^/^ *^−^* mice demonstrated dramatically decreased CXCL12 mRNA relative to CXCL12*^fl/fl^* controls (*P* = .037) (Figure S3a). When these uteri were used in our endometriosis model, lesions still grew to sizes comparable to those of CXCL12*^fl/fl^* controls (0.762 ± 0.104 mm^2^, n = 35 lesions vs 0.547 ± 0.118 mm^2^, n = 12 lesions, respectively, *P* = NS) (Figure S3b) and exhibited comparable cycle‐matched proliferation (Figure S3c), suggesting that expression of CXCL12 by the endometriosis lesion is not essential for lesion development.

### 3.5 In vitro studies on primary endometrial cells from endometriosis patients and AMD3100

To further evaluate the effect of AMD3100 directly on endometrial cells, primary endometrial cells stromal from women with endometriosis were treated with AMD3100. No significant changes in cell proliferation were observed when primary endometrial cells from endometriosis patients were treated with AMD3100 compared to untreated cells as shown in Figure S4. AMD3100 did not directly affect endometrial cells, further supporting the effect of AMD3100 specifically on BMDCs.

## 4 DISCUSSION

Interaction of CXCL12 with its G protein‐coupled receptors CXCR4 and CXCR7 plays a crucial role during embryonic development, tissue repair as well as in many pathologic processes. Activation of the CXCL12‐CXCR4‐CXCR7 signalling axis in endometriosis is associated with dysfunctional chemotaxis, increased cell proliferation, angiogenesis and reduced autophagy.^18^, ^53^ Characterization and the role of bone marrow mesenchymal stem cells in translation research has been extensively studied.^54^, ^55^, ^56^, ^57^ The present findings show that endometriosis development is associated with a significant engraftment of BMDCs, that are mostly CD45− and only a small percentage are CD31+; these data provide evidence of the relative contribution of bone marrow‐derived lineages to murine endometriosis lesion establishment and thus confirming the pivotal role of BM‐derived cells not belonging to haematopoietic system in endometriosis early growth and progression. Notably, up to 30% of BMDCs in murine endometriosis expressed CXCR4, and 25% expressed CXCR7. In addition, our model of BM transplant was designed to preserve ovarian function and oestrogen production necessary for endometriosis development. The BM transplant resulted in approximately 50% chimerism; a higher percentage of BM‐derived stem cells is likely to have engrafted the lesions as we expect that unmarked endogenous BM cells also were recruited. Given the degree of chimerism, we estimate that the number of BM‐derived cells is approximately twice that measured directly as GFP+ cells.

A number of mechanisms may explain the increased CXCL12 production and stem cell recruitment in endometriosis. Hypoxia, an hallmark of endometriosis, induces CXCL12 expression.^58^ Similarly, aberrant oestrogen signalling and progesterone resistance further amplified CXCL12‐CXCR4 axis activation in endometrial cells and endometriosis.^45^, ^46^ Targeting CXCR4 or CXCR7 using AMD3100 or CCX771, respectively, in this preclinical model of endometriosis was effective in preventing and treating ectopic lesions by affecting BM cell engraftment. We demonstrated that both compounds efficiently reduced homing of BM‐derived CD45+ and CD45− cells in endometriosis. Chronic treatment of AMD3100 efficiently blocks BM CXCR4+ cell trafficking and homing in other medical conditions^58^ and our results further support this hypothesis and extend it to endometriosis. As approved for clinical use, AMD3100 is used to block CXCR4 on BM stem cells. These cells are no longer attracted to the CXCL12 produced at high levels in bone marrow; this allows bone marrow stem cell mobilization. In a similar manner, AMD3100 also blocks chemoattraction of bone marrow stem cells to CXCL12 produced by endometriosis.

The CXCL12‐CXC4‐CXCR7 axis has a crucial role in vascular remodelling and endothelial function, including angiogenesis and vasculogenesis,^59^, ^60^ and both processes have been also described in murine endometriosis.^44^ AMD3100 and CCX771 have been described as potent anti‐angiogenesis drugs.^46^, ^61^ We showed here that both treatments reduce vessel density and VEGF‐A expression in endometriosis. We do not exclude the possibility that actions of these agents other than BMDC recruitment and engraftment may have also contributed to their ability to treat endometriosis. These drugs may also have acted directly on their receptors expressed in endometriosis. Both treatments may also exert activity by acting on endometriosis‐associated vasculature expressing CXCR4 and CXCR7. Further, not all of the actions of CXCRs are mediated through GPCR and there may be additional mechanisms driving the effects seen. We did not observe any significant changes in lesion size or volume when we used the CXCL12 knockout uterus as donor tissue compared to CXCL12 floxed mice. Elimination of local endometrial CXCL12 suggests that the effect of AMD3100 in blocking CXCL12 is not a direct effect endometrial cells; rather, bone marrow cell mobilization and recruitment to endometriosis is likely mediated by systemic CXCL12 and the impact on bone marrow‐derived cells. Confirming this observation, our in vitro studies showed that there was no significant effect of AMD3100 on endometrial stromal cells from endometriosis patients. AMD3100 likely exerts its effects on endometriosis through BMDSCs. Similarly, blocking CXCR7 by CCX771 reduces angiogenesis in vivo interrupting the pro‐angiogenic CXCL12‐CXCR7 autocrine loop exerted by activated endothelial cells.^62^ Additional studies using CXCR4 and CXCR7 knockout mouse donor tissue would clarify the role of specific cell populations in endometriosis regression following AMD3100 and CCX771 treatment.

In conclusion, in a preclinical model of endometriosis, we showed that targeting the CXCL12‐CXCR4‐CXCR7 axis blocks bone marrow‐derived stem cell recruitment. Treatment leads to regression of both new and established lesions. Bone marrow‐derived stem cell recruitment is likely necessary for endometriosis lesion maintenance, and blocking trafficking of these cells treats the disease. Both drugs exhibited similar efficacy. Clinical use will likely depend on side effect profile; the effects of prolonged use are not well characterized. In addition, these therapies may have off‐target effects on other tissues; the specificity of these treatments to endometriosis has not been evaluated in these studies. CXCR4 and CXCR7 antagonists are promising novel, non‐hormonal therapies for endometriosis.

## CONFLICT OF INTEREST

All authors declare no conflict of interest.

## AUTHOR CONTRIBUTIONS

Taylor HS, Pluchino N and Mamillapalli R critically revised the manuscript and designed this study. Pluchino N, Shaikh S and Mamillapalli R performed the acquisition, analysis and interpretation of the data: Tal A helped in CXCL12 knockout experiments while Habata S and Gaye M helped in immunofluorescence experiments. Taylor HS revised the manuscript critically and finally approved the version to be published. All authors have read and approved the final manuscript.

## Data Availability

Dr Taylor HS and Dr Mamillapalli R had full access to all of the data in the study and takes responsibility for the integrity of the data and the accuracy of the data analysis.
