# Supplementary figures and images for: CXCR4 or CXCR7 antagonists treat endometriosis by reducing bone marrow cell trafficking
Source: J Cell Mol Med. 2020 Jan 6;24(4):2464–74. doi: 10.1111/jcmm.14933 (PMC7028867; doi:10.1111/jcmm.14933)

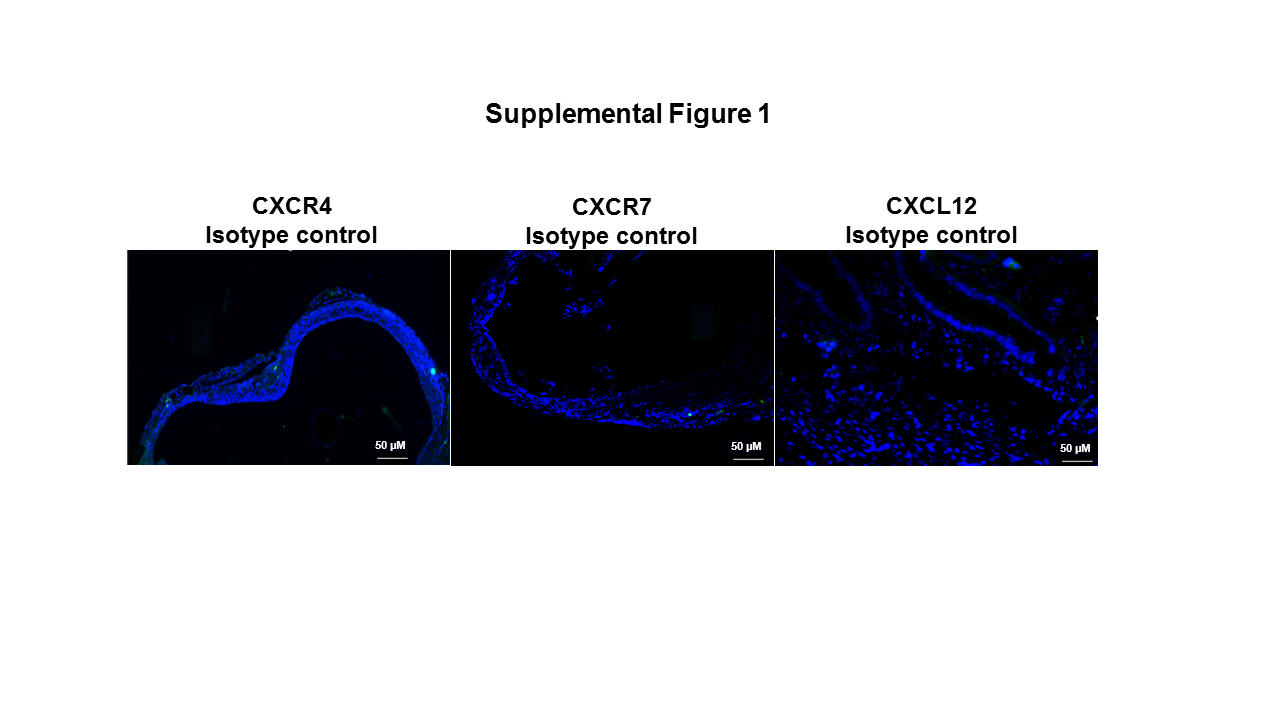

Supplement: Supplementary file 1 [file JCMM-24-2464-s001.tif]

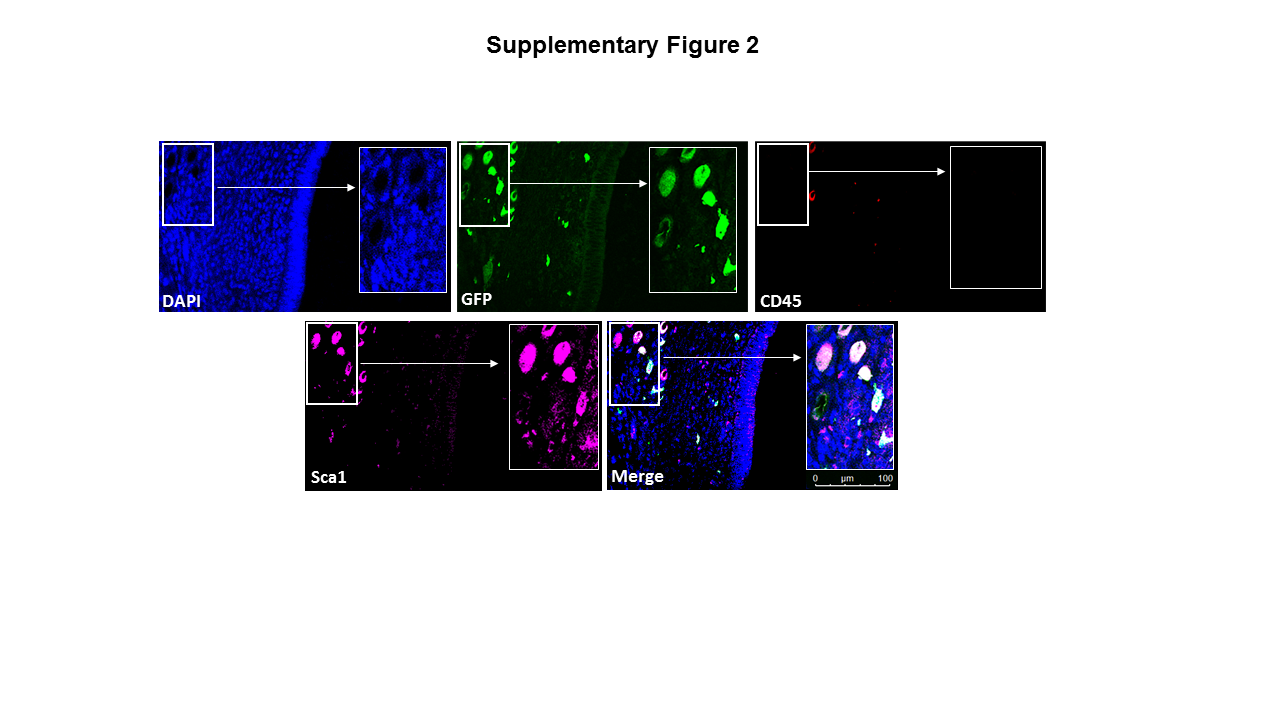

Supplement: Supplementary file 2 [file JCMM-24-2464-s002.tif]

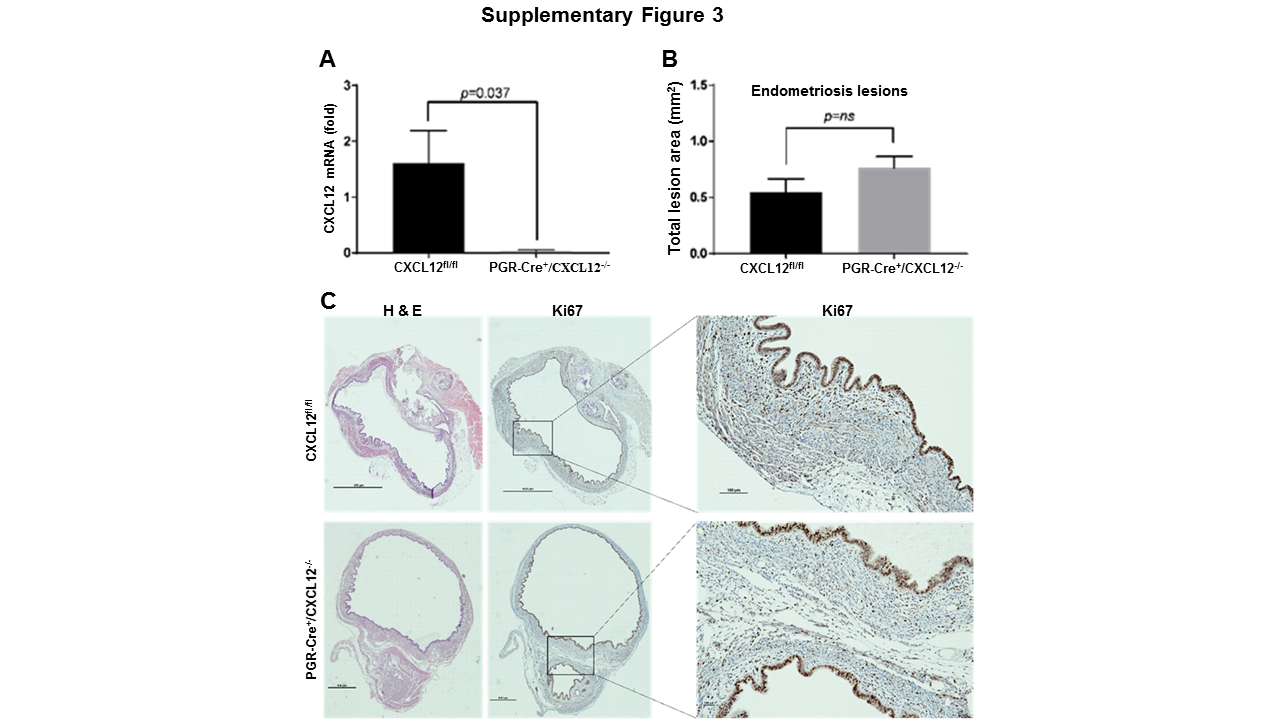

Supplement: Supplementary file 3 [file JCMM-24-2464-s003.tif]

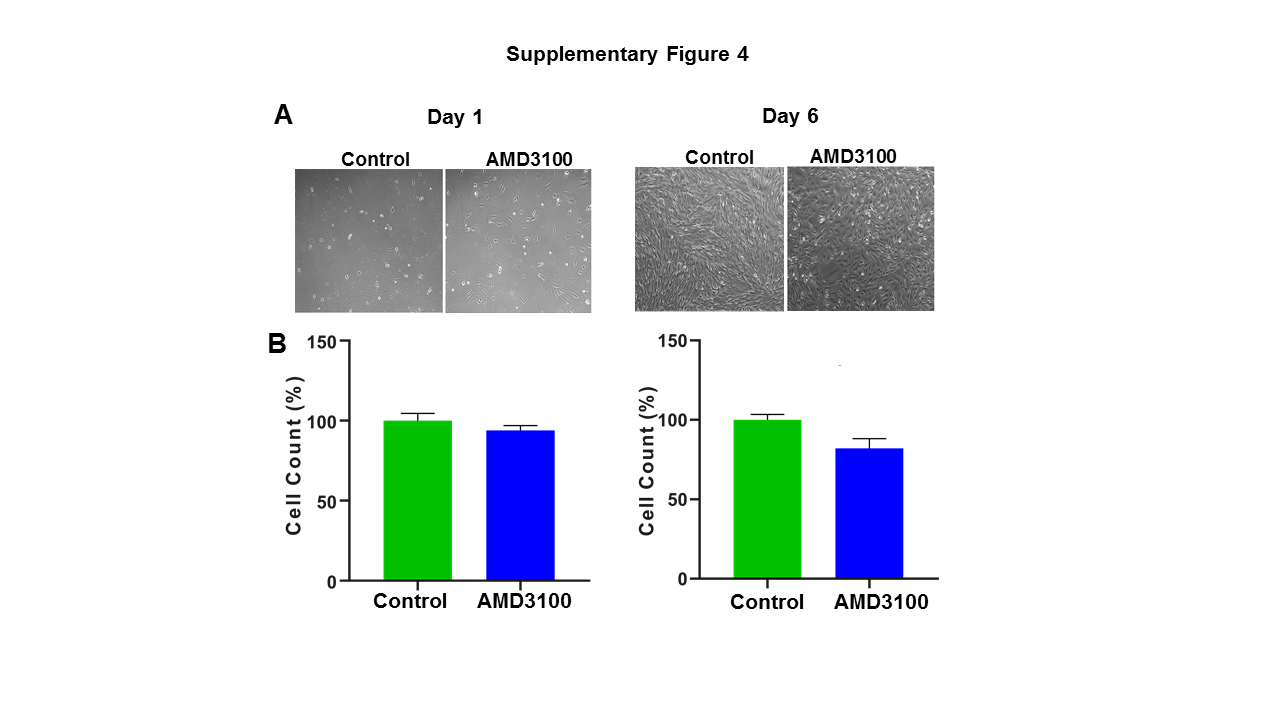

Supplement: Supplementary file 4 [file JCMM-24-2464-s004.tif]
